# Virological efficacy and emergence of drug resistance in adults on antiretroviral treatment in rural Tanzania

**DOI:** 10.1186/1471-2334-9-108

**Published:** 2009-07-07

**Authors:** Asgeir Johannessen, Ezra Naman, Sokoine L Kivuyo, Mabula J Kasubi, Mona Holberg-Petersen, Mecky I Matee, Svein G Gundersen, Johan N Bruun

**Affiliations:** 1 Ulleval Department of Infectious Diseases, Oslo University Hospital, Oslo, Norway; 2 HIV Care and Treatment Centre, Haydom Lutheran Hospital, Mbulu, Tanzania; 3 National Institute for Medical Research, Haydom Research Station, Mbulu, Tanzania; 4 Department of Microbiology and Immunology, Muhimbili National Hospital, Dar es Salaam, Tanzania; 5 Ulleval Department of Microbiology, Oslo University Hospital, Oslo, Norway; 6 Department of Microbiology and Immunology, Muhimbili University of Health and Allied Sciences, Dar es Salaam, Tanzania; 7 Research Unit, Sorlandet Hospital HF, Kristiansand, Norway; 8 Centre for Development Studies, University of Agder, Kristiansand, Norway; 9 Department of Infectious Diseases, University Hospital of North Norway, Tromso, Norway

## Abstract

**Background:**

Virological response to antiretroviral treatment (ART) in rural Africa is poorly described. We examined virological efficacy and emergence of drug resistance in adults receiving first-line ART for up to 4 years in rural Tanzania.

**Methods:**

Haydom Lutheran Hospital has provided ART to HIV-infected patients since October 2003. A combination of stavudine or zidovudine with lamivudine and either nevirapine or efavirenz is the standard first-line regimen. Nested in a longitudinal cohort study of patients consecutively starting ART, we carried out a cross-sectional virological efficacy survey between November 2007 and June 2008. HIV viral load was measured in all adults who had completed at least 6 months first-line ART, and genotypic resistance was determined in patients with viral load >1000 copies/mL.

**Results:**

Virological response was measured in 212 patients, of whom 158 (74.5%) were women, and median age was 35 years (interquartile range [IQR] 29–43). Median follow-up time was 22.3 months (IQR 14.0–29.9). Virological suppression, defined as <400 copies/mL, was observed in 187 patients (88.2%). Overall, prevalence of ≥1 clinically significant resistance mutation was 3.9, 8.4, 16.7 and 12.5% in patients receiving ART for 1, 2, 3 and 4 years, respectively. Among those successfully genotyped, the most frequent mutations were M184I/V (64%), conferring resistance to lamivudine, and K103N (27%), Y181C (27%) and G190A (27%), conferring resistance to non-nucleoside reverse transcriptase inhibitors (NNRTIs), whereas 23% had thymidine analogue mutations (TAMs), associated with cross-resistance to all nucleoside reverse transcriptase inhibitors (NRTIs). Dual-class resistance, i.e. resistance to both NRTIs and NNRTIs, was found in 64%.

**Conclusion:**

Virological suppression rates were good up to 4 years after initiating ART in a rural Tanzanian hospital. However, drug resistance increased with time, and dual-class resistance was common, raising concerns about exhaustion of future antiretroviral drug options. This study might provide a useful forecast of drug resistance and demand for second-line antiretroviral drugs in rural Africa in the coming years.

## Background

Access to antiretroviral treatment (ART) of HIV/AIDS has increased substantially over the past few years throughout the developing world. Lower prices of antiretroviral drugs combined with political determination have given rise to one of the greatest public health operations of our time, spearheaded by World Health Organization (WHO), Joint United Nations Programme on HIV/AIDS (UNAIDS) and international non-governmental organizations (NGOs). By December 2007, three million people were receiving ART in low- and middle-income countries, but still this was only 31% of those estimated to be in need of it [1].

ART programs in developing countries are now moving from early pioneer projects to a sustained effort. Inevitably, the long-term challenges of providing ART will become increasingly evident, including late drug toxicities, treatment failure and emergence of drug resistance [2-4]. Indeed, some have argued that scaling up ART in Africa could create widespread drug resistance [5,6]. Early reports, however, have documented good adherence to therapy [7] and short-term virological efficacy comparable to industrialized countries [8].

Although several studies on ART efficacy in Africa have been published, the majority have been carried out in larger cities [9-11], often with NGO support [10,12], and usually with short follow-up time [9,10,12]. However, the majority of Africans reside in rural areas [13], and little is known about the long-term effects of ART in such settings. The key to long-term benefit of ART is sustained suppression of viral replication and avoidance of resistance [14-16]. Our aim was to assess virological efficacy and emergence of drug resistance in HIV-infected patients up to 4 years after starting first-line ART in a rural Tanzanian hospital.

## Methods

### Study setting, participants and treatment

Tanzania is a low-income country with an estimated HIV prevalence of 6.2% [1]. The National AIDS Control Program started to scale up antiretroviral treatment from 2005, and by December 2007, 135,696 people were receiving ART [1]. Haydom Lutheran Hospital is a 400-bed hospital in Manyara region owned by the Evangelical Lutheran Church of Tanzania. It is the main health care provider to a rural population of about 260,000 people. In 2002, the hospital launched a comprehensive HIV prevention and intervention program, which has previously been described in detail [17]. In brief, free treatment and care has been offered to all HIV-infected patients since October 2003, including free drugs and in-patient care. Clinical officers have been trained by experienced HIV physicians to treat and follow-up patients. The HIV program in Haydom is now integrated in the National AIDS Control Program.

All patients were assessed with a standardized evaluation form at enrolment, where demographic data, medical history, clinical findings and laboratory investigations were recorded. ART was initiated in accordance with WHO's recommendations [18-20]: WHO stage 4 irrespective of CD4 cell count, WHO stage 3 with CD4 ≤350 cells/μL, or CD4 ≤200 cells/μL with any WHO stage. However, reliable CD4 cell counts were not available until September 2006; thus, most patients started ART based on clinical criteria only (WHO stage 3 and 4). In addition, triple-drug combination ART, and not single-dose nevirapine, was offered to HIV-infected pregnant and lactating women, from pregnancy week 20 till cessation of breast-feeding, irrespective of WHO stage and CD4 cell count, to prevent mother-to-child transmission (PMTCT).

First-line treatment was stavudine or zidovudine, combined with lamivudine, and either nevirapine or efavirenz. A generic fixed-dose combination of stavudine, lamivudine and nevirapine was preferred whenever possible. Patients with CD4 ≤200 cells/μL or WHO stage 3 or 4 disease were given co-trimoxazole prophylaxis 960 mg thrice weekly. Second-line treatment was available from December 2006 and comprised lopinavir/ritonavir, didanosine and abacavir. Criteria for switching to second-line ART was virological failure as recommended by WHO (i.e. >10,000 copies/mL) [20]; however, viral load was not measured routinely, and only selected patients with high clinical suspicion of failure were tested.

Nested in a longitudinal cohort study of patients consecutively starting ART, we carried out a cross-sectional virological efficacy survey between November 15, 2007 and June 6, 2008. All adults (≥15 years) who had received first-line ART for more than 6 months were considered eligible. Patients were included regardless of previous adherence or treatment interruptions. However, those who had stopped ART for ≥1 month and not re-started at the time of the survey were classified as "stopped treatment" and excluded. Furthermore, those who had already switched to second-line ART were excluded since genotypic resistance results prior to the switch were unavailable. Ethical approval was granted by National Institute for Medical Research in Tanzania and Regional Committee for Medical Research Ethics in Norway, and all patients gave written consent to participate in the study.

### Laboratory investigations

Standard laboratory investigations at baseline included: Full blood cell count, erythrocyte sedimentation rate, liver function tests, creatinine, blood sugar, hepatitis B surface antigen and syphilis serology. Patients who started ART were followed up with laboratory investigations every three months. Hematology was measured using the Sysmex KX-21 Hematology Analyzer (Sysmex Corp., Kobe, Japan). CD4 cell counts were available from September 2006 using the FACSCount flow cytometer (Becton Dickinson, San Jose, California, USA).

Plasma specimens for virological analyses were stored at -20°C until shipment to the reference laboratory. Manufacturer's instructions were followed with regard to sample collection and transport. HIV viral load was measured at Muhimbili National Hospital, Dar es Salaam, Tanzania, using the Cobas TaqMan 48 Analyzer (Roche Diagnostics, Branchburg, New Jersey, USA) with a lower detection limit at 40 copies/mL; however, due to equipment breakdown, one third of the samples were analysed with the Cobas Amplicor HIV-1 Monitor v1.5 (Roche Diagnostics, Branchburg, New Jersey, USA) with a detection limit at 400 copies/mL. All specimens with viral load >1000 copies/mL were sent to Ulleval University Hospital, Oslo, Norway, for genotypic resistance testing. The ViroSeq HIV-1 Genotyping System (Abbott Molecular, De Plains, Illinois, USA) was used to determine HIV-1 subtype and mutations in the protease and reverse transcriptase genes. Only drug resistance mutations listed in the Spring 2008 update from the International AIDS Society were considered [21]. Resistance profiles to antiretroviral drugs were interpreted according to the Stanford University HIV Drug Resistance Database (HIVdb Program, http://hivdb.stanford.edu).

### Statistical analysis

The main outcomes of interest were on-treatment virological suppression and clinically significant genotypic resistance. Virological suppression was defined as HIV viral load <400 copies/mL, since this was the detection limit of the least sensitive assay used in this study. Clinically significant genotypic resistance was defined as HIV viral load >1000 copies/mL and presence of ≥1 drug resistance mutation listed in the Spring 2008 update from the International AIDS Society [21]. Duration of ART was rounded off to the nearest full year (1, 2, 3 or 4 years) when presenting prevalence of virological suppression and drug resistance. Logistic regression was used to study associations between baseline characteristics and emergence of drug resistance. Univariable analysis was performed for the following variables: Sex, age, WHO stage, initial ART regimen, duration of ART, body mass index, hemoglobin level and total lymphocyte count. CD4 cell counts were excluded because of too few observations. Variables with *P *< 0.1 in univariable analyses were advanced into a multivariable regression analysis, using the forward stepwise (Wald) method to avoid overcorrection. Multicollinearity was excluded using Spearman's correlation coefficient with a cutoff at 0.7. Data were analysed with SPSS version 16.0 for Windows (SPSS Inc., Chicago, Illinois, USA), except 95% confidence intervals (CI) for proportions which were calculated with NCSS version 2007 (NCSS, Kaysville, Utah, USA). All tests were two-sided and level of significance was set at *P *< 0.05.

## Results

### Baseline characteristics

**Figure 1:**
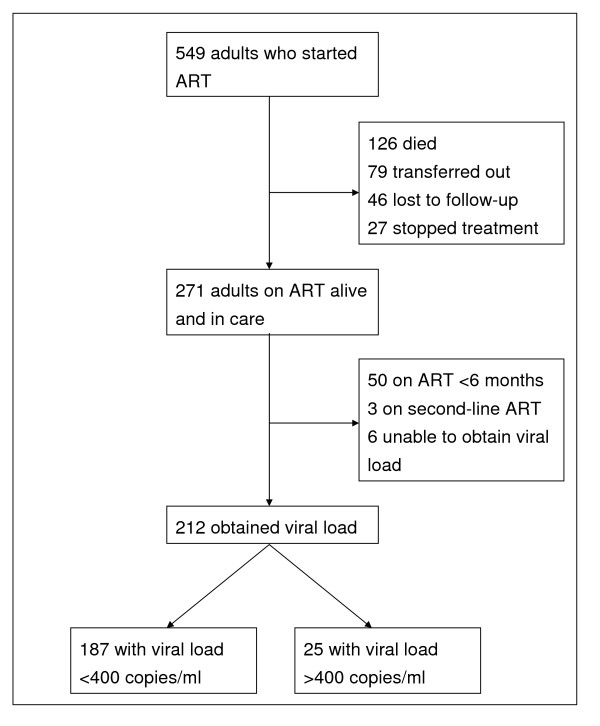
**Profile of the study cohort, Haydom Lutheran Hospital, Tanzania, 2003–08**.

Out of 549 adults who enrolled in the HIV program and started ART, 126 patients (23.0%) died, of whom 76 died within 3 months of starting ART. Seventy-nine patients (14.4%) were transferred to another health facility, 46 (8.4%) were lost to follow-up, whereas 27 patients (4.9%) self-stopped treatment after receiving ART for a median of 11.3 months. Fifty patients were not eligible for the virological survey because they had taken ART for less than 6 months, and 3 because they were on second-line ART. Among the remaining 218 patients who were selected for the survey, plasma was obtained from 212 of them. Six patients failed to participate due to: Temporary travel to another area (*n *= 3), error in specimen preparation (*n *= 1) or unknown (*n *= 2). The study profile is presented in figure 1.

Median follow-up time among 212 patients included in this study was 22.3 months (interquartile range [IQR] 14.0–29.9). Median age was 35 years (IQR 29–43), and 158 patients (74.5%) were women. At the time of ART initiation, 110 (52.1%) had clinical AIDS (WHO stage 4). The most common AIDS defining events were: Wasting syndrome (87.3%), oesophageal candidiasis (10.9%), extrapulmonary tuberculosis (4.5%) and Kaposi's sarcoma (4.5%). Initial ART regimen was stavudine/lamivudine/nevirapine in 122 patients (57.5%), stavudine/lamivudine/efavirenz in 39 (18.4%), zidovudine/lamivudine/nevirapine in 45 (21.2%), and zidovudine/lamivudine/efavirenz in 6 (2.8%). Among 66 patients with a baseline CD4 measurement, median CD4 cell count was 118 cells/μL (IQR 51–189).

### Virological results

**Figure 2:**
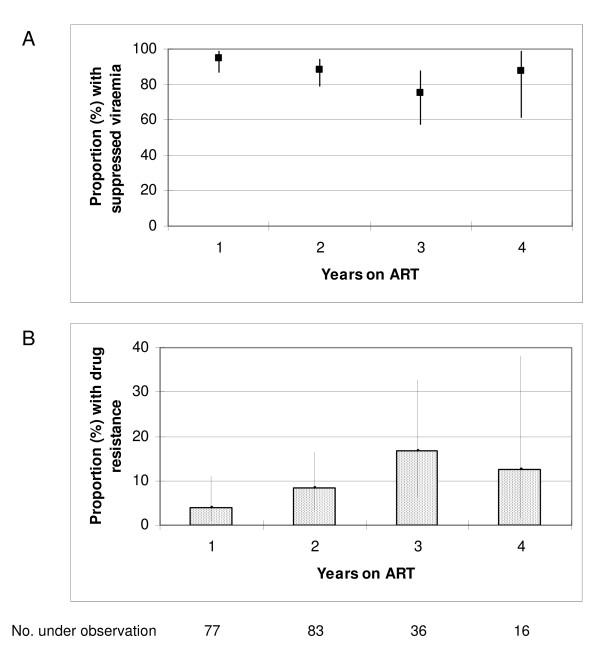
**Proportion of patients on ART with: A) suppressed viraemia (<400 copies/mL), and B) ≥1 clinically significant resistance mutation**. Vertical lines indicate 95% confidence interval.

Overall, 187 patients (88.2%; 95% CI 83.1–92.2) had suppressed viraemia (<400 copies/mL). Two patients (0.9%) had 400–1000 copies/mL, 14 (6.6%) had 1000–10,000 copies/mL, 5 (2.4%) had 10,000–100,000 copies/mL, and 4 (1.9%) had >100,000 copies/mL. The proportion of patients (95% CI) with suppressed viraemia after 1, 2, 3 and 4 years was 94.8% (87.2–98.6), 88.0% (79.0–94.1), 75.0% (57.8–87.9) and 87.5% (61.7–98.4), respectively (figure 2). The small number of patients who received ART for 3 and 4 years (n = 36 and n = 16, respectively) gave rise to wide confidence intervals for those groups.

Genotyping was successful in 22 of 23 samples with viral load >1000 copies/mL. HIV-1 subtypes were A (*n *= 3), C (*n *= 7), D (*n *= 8) and CRF01_AE (*n *= 2), whereas 2 were inconclusive (different subtypes in the protease and reverse transcriptase genes). Among those successfully genotyped, 18 patients (82%) harboured at least one clinically relevant resistance mutation in the reverse transcriptase gene (table 1). The most frequent mutations were M184I/V (*n *= 14; 64%), conferring resistance to lamivudine, and K103N (*n *= 6; 27%), Y181C (*n *= 6; 27%) and G190A (*n *= 6; 27%), conferring resistance to non-nucleoside reverse transcriptase inhibitors (NNRTIs). Thymidine analogue mutations (TAMs), associated with cross-resistance to all nucleoside reverse transcriptase inhibitors (NRTIs), were found in 5 patients (23%), of whom 1 had ≥3 TAMs. Fourteen patients (64%) had dual-class resistance, i.e. resistance to both NRTIs and NNRTIs. None had clinically relevant mutations in the protease gene.

**Table 1:** Genotypic resistance results in 23 patients on ART with virological failure (HIV-1 RNA >1000 copies/mL)

ID	Sex	Age	ART regimen: initial (current^a^)	Months on ART	Subtype	Viral load	Protease mutations	Reverse transcriptase mutations
34	M	24	ZDV/3TC/NVP (ZDV/3TC/EFV)	42.1	C	434,131	M36I, L63P, H69K, I93L	K103N
84	M	30	d4T/3TC/EFV (d4T/3TC/NVP)	49.1	D	8690	I13V, L33V, M36I, I64V	K103N, M184V
224	M	32	d4T/3TC/NVP	34.7	C	1349	M36I, L63P, H69K, I93L	
240	M	43	d4T/3TC/EFV (d4T/3TC/NVP)	35.5	CRF01_AE	81,691	I13V, M36I, L63P, H69K	D67N, K70R, K103N, V179T, M184V, K219Q
275	F	41	d4T/3TC/NVP	32.3	D	477,518	I13V, K20R, M36V, L63P, I64V, I93L	G190A
282	F	32	d4T/3TC/NVP	32.4	A	2621	L10I, I13V, M36I, H69K	M184V, Y188C
307	F	35	d4T/3TC/NVP (d4T/3TC/EFV)	31.7	*Failed*	2301	*Failed*	*Failed*
321	F	34	d4T/3TC/NVP	30.4	D	1504	I13V, K20R, M36I, I62V, I64V	Y181C, M184I
366	F	48	d4T/3TC/NVP	28.3	C	3000	M36I, H69K, I93L	K103N, V179T, M184V
401	F	44	d4T/3TC/NVP	28.6	C	2290	M36I, D60E, H69K, I93L	G190A
402	F	40	d4T/3TC/NVP (d4T/3TC/EFV)	28.3	?^b^	20,500	L10I, I13V, G16E, M36I, H69K	K101P, M184V, G190A, T215F
410	F	45	d4T/3TC/NVP	36.9	D	5990	I13V, L63P, I64V, V77I	K101E, M184V, G190A
473	F	35	d4T/3TC/NVP	27.3	D	3965	I13V, I64V, V77I	M41L, V75I, Y181C, M184V
476	F	19	ZDV/3TC/NVP (d4T/3TC/NVP)	26.5	D	1980	I64V	G190A
516	F	24	d4T/3TC/EFV	24.0	C	419,979	M36I, L63P, H69K, I93L	
554	F	28	ZDV/3TC/NVP (d4T/3TC/NVP)	24.0	C	1886	K20R, M36I, H69K	
583	F	30	d4T/3TC/NVP	21.1	CRF01_AE	1432	L10V, G16E, M36IV, H69K	K103N, Y181C, M184V
611	F	33	d4T/3TC/NVP	17.8	C	15,600	M36I, I62V, L63P, H69K, V82I, I93L	K65R, V75I, V108I, Y181C, M184V, L210W
643	F	37	d4T/3TC/NVP	31.3	D	2400	K20R, M36I, I62V, I64M	K70R, Y181C, M184V
749	F	32	d4T/3TC/EFV (d4T/3TC/NVP)	19.0	?^c^	35,400	I13V, M36I, H69K	K103N, M184V
752	F	35	d4T/3TC/NVP	14.5	A	3101	L10I, M36I, H69K	Y181C, M184V
785	F	26	d4T/3TC/NVP (d4T/3TC/EFV)	14.6	D	3,683,117	I13V, M36I, D60E, I64V	
982	F	15	d4T/3TC/NVP	8.5	A	76,700	I13V, M36I, L63P, H69K, V82I	V179T, M184V, G190A

^a^Only specified if different from initial regimen.^b^CRF_01AE in the protease and A in the reverse transcriptase gene. ^c^A in the protease and CRF_01AE in the reverse transcriptase gene.

ART, antiretroviral treatment; d4T, stavudine; 3TC, lamivudine; NVP, nevirapine; EFV, efavirenz; ZDV, zidovudine.

Hence, in total, 18 of 212 patients (8.5%; 95% CI 5.1–13.1) harboured drug resistance by use of a standard genotyping assay. The prevalence (95% CI) of any clinically significant drug resistance after 1, 2, 3 and 4 years was 3.9% (0.8–11.0), 8.4% (3.5–16.6), 16.7% (6.4–32.8) and 12.5% (1.6–38.3), respectively (figure 2). Dual-class resistance was observed in 3.9% (0.8–11.0), 6.0% (2.0–13.5), 13.9% (4.7–29.5) and 6.3% (0.2–30.2), respectively. Again, the small number of patients on ART for 3 and 4 years gave rise to wide confidence intervals.

### Predictors of drug resistance

In univariable logistic regression analysis only duration of ART was significantly associated with emergence of drug resistance (≥3 years on ART; odds ratio [OR] 4.49; 95% CI 1.13–17.8; *P *= 0.033). Anemia (hemoglobin <10 g/dL; OR 2.84; 95% CI 0.97–8.32; *P *= 0.058) and lymphopenia (total lymphocyte count <1.2 × 10^9^/L; OR 2.91; 95% CI 0.99–8.53; *P *= 0.052) were borderline significant. No associations were found for age, sex, clinical stage, body mass index or initial ART regimen. In multivariable analysis where duration of ART, anemia and lymphopenia were included using the forward stepwise method, only duration of ART remained in the final model, with the same odds ratio and *P*-value as above (table 2).

**Table 2:** Predictors of drug resistance in 212 HIV-infected adults on ART in rural Tanzania

	Univariable^a^		Multivariable^b^	
		
Predictor variables	OR (95% CI)	*P*	OR (95% CI)	*P*
Duration of ART				
1 year	1		1	
2 years	2.27 (0.57–9.12)	0.247	2.27 (0.57–9.12)	0.247
≥ 3 years	4.49 (1.13–17.8)	0.033	4.49 (1.13–17.8)	0.033
Hemoglobin				
≥ 10 g/dL	1			
<10 g/dL	2.84 (0.97–8.32)	0.058	NS	
Total lymphocyte count				
≥ 1.2 × 10^9^/L	1			
<1.2 × 10^9^/L	2.91 (0.99–8.53)	0.052	NS	

^a ^No significant associations were found for age, sex, clinical stage, body mass index or initial ART regimen.

^b^Stepwise multivariable logistic regression using a forward selection procedure. NS denotes that the variable did not meet the significance level criterion (*P *<0.05) for inclusion in the final model.

OR, odds ratio; CI, confidence interval; ART, antiretroviral therapy.

Two patients with low-level viraemia (400–1000 copies/mL) and one patient whose genotyping failed were assumed not to harbour resistance in our study. To assess whether this assumption might have biased our results, we conducted a sensitivity analysis where these patients were classified as resistant. In the resulting multivariable model, both duration of ART (≥3 years on ART; OR 6.47; 95% CI 1.28–32.6; *P *= 0.024) and lymphopenia (total lymphocyte count <1.2 × 10^9^/L; OR 4.24; 95% CI 1.48–12.2; *P *= 0.007) were significantly associated with resistance. Hence, the effect of lymphopenia might have been underestimated by misclassification bias in the main analysis, whereas duration of ART was a strong and significant predictor of resistance in both analyses.

## Discussion

Virological suppression rates were good up to 4 years after starting ART in a rural Tanzanian hospital. These results are in keeping with early reports from resource-limited settings, where short-term virological efficacy rates were as good as those reported from Europe and North America [8]. We show that suppression of viraemia can be sustained for several years even in rural Africa, where logistical support is challenging and patients often live in poverty. However, like in many other African ART programs the attrition rate was high, and strategies to reduce early mortality and other program losses need to be identified [11,12,22,23].

Experiences elsewhere have shown that poorly managed HIV programs can give rise to widespread drug resistance [24]. A number of factors may have contributed to the sustained virological efficacy of the ART program in the present study. First, all treatment and care for HIV-infected patients was provided free of charge, which has previously been shown to improve treatment efficacy [8]. Second, all patients had three days of adherence counselling with a nurse prior to starting ART. Third, a close collaboration between the clinical staff and a network of community home-based carers ensured follow-up of patients in their villages. Fourth, regular educational peer-support meetings contributed to reduce the stigma and isolation many patients experience after receiving an HIV diagnosis. Fifth, antiretroviral drug supply continuity was uninterrupted from the beginning of the program. And finally, visiting HIV physicians focused on capacity building of local clinical officers, with emphasis on common curable opportunistic infections, such as tuberculosis, candidiasis, cryptococcal meningitis and cerebral toxoplasmosis.

Overall, emergence of drug resistance was relatively uncommon; only 8.5% harboured clinically significant resistance mutations. Although the proportion of patients with drug resistance was low, however, in a high-prevalence country like Tanzania the absolute number of individuals in need of second-line ART can rapidly become substantial. If our results were extrapolated to Tanzania as of December 2007 [1], then 11,500 patients would harbour drug resistance and be in need of second-line ART. Such an extrapolation is not necessarily valid, but it illustrates the magnitude of the problem that drug resistance can inflict on national ART programs. The number of individuals receiving ART has increased 20-fold over the past 4 years in sub-Saharan Africa [1], but access to second-line antiretroviral drugs is still limited in many developing countries due to higher costs and lack of fixed-dose combinations. Our study underscores the growing global need for affordable and convenient second-line antiretroviral regimens.

The prevalence of drug resistance increased with time and reached approximately 15% after 3–4 years on ART. Most previous studies on ART in Africa have focused on early treatment efficacy, showing good virological results with a limited observation time [10,12,25]. Only a few studies have assessed long-term (>2 years) emergence of drug resistance in sub-Saharan Africa. An early study from Senegal showed that 12.5% had one or more drug resistance mutations after a median of 30 months on ART [26], whereas a recent study from Côte d'Ivoire found 22% resistance after a median of 37 months on ART [27]. These results should not be used as an argument against HIV treatment in Africa; in fact, the results are comparable to a recent study from Canada, where 20% developed resistance after 30 months on the ART regimen most widely used in resource-limited settings (stavudine/lamivudine/nevirapine) [28]. Thus, emergence of drug resistance appears to occur at a similar rate in Africa as in a Western setting.

Of concern, among 18 patients with drug resistance mutations, 14 harboured dual-class resistance. All 14 had a combination of M184I/V, conferring resistance to lamivudine, with one or more of K103N, Y181C and G190A, conferring resistance to NNRTIs. Five of these patients also had thymidine analogue mutations (TAMs), associated with cross-resistance to all NRTIs. In 3 patients the standard second-line regimen in Tanzania would not be adequate, i.e. would not introduce at least 2 fully active drugs, which is the recommended strategy in treatment failure [29]. Other studies from low- and middle-income countries, using the same first-line treatment, have found a similar pattern. In a recent study from Angola, 65% of patients with virological failure had dual-class resistance [30]. Furthermore, a study from Thailand found that second-line treatment options, in the absence of newer antiretroviral drugs, were limited for 48% of patients failing their initial regimen [31]. Expanding access to newer antiretroviral drugs, including new HIV drug classes, should be a priority in the global efforts to control HIV/AIDS.

It has been shown that in the presence of a failing ART regimen, resistance mutations accumulate, jeopardizing future treatment options [32]. Early detection of treatment failure rely on viral load measurements, a standard component of ART programs in resource-rich countries [29]. In the present program, like in most resource-limited settings, viral load was not measured routinely, and treatment failure had to be assessed by clinical signs and CD4 cell counts. However, clinical signs and CD4 decline, as recommended by WHO to detect treatment failure in the absence of viral loads, have poor sensitivity and specificity, and result in frequent misclassifications [33,34]. Hence, there is an urgent need for a simple, affordable viral load assay adapted for use in basic, tropical environments, so that treatment failure can be detected before multiple mutations occur.

In our study only duration of ART was significantly associated with emergence of drug resistance. Baseline anemia and lymphopenia were borderline significant in univariable analysis, and our sample size might have been too small to reveal a true association. Other studies have found that low CD4 cell count and high viral load at baseline increase the risk of drug resistance [35]; however, these measurements were not available in our study.

There were some weaknesses of this study. First, virological efficacy could only be assessed in patients who were alive and in care. A high early mortality accounted for most of the program loss, which probably reflected advanced immunodeficiency at enrolment rather than treatment failure [17]. Many patients were transferred out when the National AIDS Control Program started scaling up ART in other villages, but it is unlikely that this introduced any systematic bias. Among patients who stopped treatment (4.9%) or were lost to follow-up (8.4%), there was probably a proportion who either died or developed drug resistance. Our study must be considered an "on-treatment" analysis, and virological suppression rates and resistance estimates in an "intention-to-treat" analysis would have been poorer. Another limitation of this study was the lack of longitudinal viral load and resistance results. A cross-sectional virological survey may be more influenced by random biological variations and laboratory artefacts, being derived from a single time point. Also, we can not ascertain whether drug resistance mutations existed prior to initiation of ART, which was recently observed in rural South Africa [36], or developed during treatment. However, Haydom Lutheran Hospital was the first ART provider in the area, and single-dose nevirapine was not used for PMTCT, so it is unlikely that there was any significant primary resistance. Furthermore, this study was limited by lack of adherence data, which is considered the most important predictor of resistance [35]; however, adherence estimates would not have altered our conclusions. Finally, this was a hospital based study and probably there was a selection bias towards more advanced immunodeficiency at baseline, which has previously been shown to increase the risk of drug resistance [35]. On the other hand, late presentation has been observed in many African ART programs [10-12,25,37,38], and we believe our findings can be representative of other similar settings.

## Conclusion

We found good virological suppression rates up to 4 years after initiating ART in a rural hospital in Tanzania. These results suggest that ART can be safely scaled up in rural Africa with similar long-term virological efficacy rates as those reported for industrialized countries. However, prevalence of drug resistance increased with time, and dual-class resistance was common, raising concerns about exhaustion of future antiretroviral drug options. Earlier detection of treatment failure and timely switch to second-line ART could reduce accumulation of drug resistance, underscoring the growing need for virological monitoring in resource-limited settings. This study might provide a useful forecast of drug resistance and demand for second-line antiretroviral drugs in rural Africa in the coming years.

## Competing interests

The authors declare that they have no competing interests.

## Authors' contributions

AJ analyzed the data and drafted the manuscript. EN collected the data. MJK and MH-P were responsible for the laboratory analyses. MIM participated in the conception of the study. SLK participated in the data collection and coordination of the study. SGG and JNB conceived the study, and participated in its design and coordination. All authors read and approved the final manuscript.

## Pre-publication history

The pre-publication history for this paper can be accessed here:

http://www.biomedcentral.com/1471-2334/9/108/prepub

## References

[B1] Joint United Nations Programme on HIV/AIDS (UNAIDS) Report on the Global AIDS Epidemic 2008 2008 Geneva: UNAIDS

[B2] Sukasem C Churdboonchart V Chasombat S Kohreanudom S Watitpun C Pasomsub E Surveillance of genotypic resistance mutations in chronic HIV-1 treated individuals after completion of the National Access to Antiretroviral Program in Thailand Infection 2007 35 81 88 10.1007/s15010-007-6169-x 17401711

[B3] Marconi VC Sunpath H Lu Z Gordon M Koranteng-Apeagyei K Hampton J Prevalence of HIV-1 drug resistance after failure of a first highly active antiretroviral therapy regimen in KwaZulu Natal, South Africa Clin Infect Dis 2008 46 1589 1597 18419495 10.1086/587109 PMC2692213

[B4] van Griensven J De Naeyer L Mushi T Ubarijoro S Gashumba D Gazille C High prevalence of lipoatrophy among patients on stavudine-containing first-line antiretroviral therapy regimens in Rwanda Trans R Soc Trop Med Hyg 2007 101 793 798 10.1016/j.trstmh.2007.02.020 17467756

[B5] Harries AD Nyangulu DS Hargreaves NJ Kaluwa O Salaniponi FM Preventing antiretroviral anarchy in sub-Saharan Africa Lancet 2001 358 410 414 10.1016/S0140-6736(01)05551-9 11502341

[B6] Popp D Fisher JD First, do no harm: a call for emphasizing adherence and HIV prevention interventions in active antiretroviral therapy programs in the developing world AIDS 2002 16 676 678 10.1097/00002030-200203080-00025 11873017

[B7] Mills EJ Nachega JB Buchan I Orbinski J Attaran A Singh S Adherence to antiretroviral therapy in sub-Saharan Africa and North America: a meta-analysis JAMA 2006 296 679 690 10.1001/jama.296.6.679 16896111

[B8] Ivers LC Kendrick D Doucette K Efficacy of antiretroviral therapy programs in resource-poor settings: a meta-analysis of the published literature Clin Infect Dis 2005 41 217 224 10.1086/431199 15983918

[B9] Kamya MR Mayanja-Kizza H Kambugu A Bakeera-Kitaka S Semitala F Mwebaze-Songa P Predictors of long-term viral failure among ugandan children and adults treated with antiretroviral therapy J Acquir Immune Defic Syndr 2007 46 187 193 10.1097/QAI.0b013e31814278c0 17693883

[B10] Coetzee D Hildebrand K Boulle A Maartens G Louis F Labatala V Outcomes after two years of providing antiretroviral treatment in Khayelitsha, South Africa AIDS 2004 18 887 895 10.1097/00002030-200404090-00006 15060436

[B11] Bussmann H Wester CW Ndwapi N Grundmann N Gaolathe T Puvimanasinghe J Five-year outcomes of initial patients treated in Botswana's National Antiretroviral Treatment Program AIDS 2008 22 2303 2311 10.1097/QAD.0b013e3283129db0 18981769 PMC2853026

[B12] Ferradini L Jeannin A Pinoges L Izopet J Odhiambo D Mankhambo L Scaling up of highly active antiretroviral therapy in a rural district of Malawi: an effectiveness assessment Lancet 2006 367 1335 1342 10.1016/S0140-6736(06)68580-2 16631912

[B13] UNFPA State of World Population 2007. Unleashing the Potential of Urban Growth 2007 New York: UNFPA

[B14] Hogg RS Bangsberg DR Lima VD Alexander C Bonner S Yip B Emergence of drug resistance is associated with an increased risk of death among patients first starting HAART PLoS Med 2006 3 e356 16984218 10.1371/journal.pmed.0030356 PMC1569883

[B15] Grabar S Le Moing V Goujard C Egger M Leport C Kazatchkine MD Response to highly active antiretroviral therapy at 6 months and long-term disease progression in HIV-1 infection J Acquir Immune Defic Syndr 2005 39 284 292 10.1097/01.qai.0000160925.33935.72 15980687

[B16] Raboud JM Montaner JS Conway B Rae S Reiss P Vella S Suppression of plasma viral load below 20 copies/mL is required to achieve a long-term response to therapy AIDS 1998 12 1619 1624 10.1097/00002030-199813000-00008 9764780

[B17] Johannessen A Naman E Ngowi BJ Sandvik L Matee MI Aglen HE Predictors of mortality in HIV-infected patients starting antiretroviral therapy in a rural hospital in Tanzania BMC Infect Dis 2008 8 52 18430196 10.1186/1471-2334-8-52 PMC2364629

[B18] WHO Scaling up antiretroviral therapy in resource-limited settings. Guidelines for a public health approach 2002 Geneva: WHO 12154788

[B19] WHO Scaling up antiretroviral therapy in resource-limited settings: Treatment guidelines for a public health approach. 2003 revision 2004 Geneva: WHO 12154788

[B20] WHO Antiretroviral therapy for HIV infection in adults and adolescents: Recommendations for a public health approach. 2006 revision 2006 Geneva: WHO 23741771

[B21] Johnson VA Brun-Vezinet F Clotet B Gunthard HF Kuritzkes DR Pillay D Update of the Drug Resistance Mutations in HIV-1: Spring 2008 Top HIV Med 2008 16 62 68 18441382 10.1007/s11750-007-0034-z

[B22] Weidle PJ Malamba S Mwebaze R Sozi C Rukundo G Downing R Assessment of a pilot antiretroviral drug therapy programme in Uganda: patients' response, survival, and drug resistance Lancet 2002 360 34 40 10.1016/S0140-6736(02)09330-3 12114039

[B23] Toure S Kouadio B Seyler C Traore M koury-Dogbo N Duvignac J Rapid scaling-up of antiretroviral therapy in 10,000 adults in Cote d'Ivoire: 2-year outcomes and determinants AIDS 2008 22 873 882 10.1097/QAD.0b013e3282f768f8 18427206 PMC3921665

[B24] Richard N Juntilla M Abraha A Demers K Paxinos E Galovich J High prevalence of antiretroviral resistance in treated Ugandans infected with non-subtype B human immunodeficiency virus type 1 AIDS Res Hum Retroviruses 2004 20 355 364 10.1089/088922204323048104 15157354

[B25] Wester CW Kim S Bussmann H Avalos A Ndwapi N Peter TF Initial response to highly active antiretroviral therapy in HIV-1C-infected adults in a public sector treatment program in Botswana J Acquir Immune Defic Syndr 2005 40 336 343 10.1097/01.qai.0000159668.80207.5b 16249709

[B26] Laurent C Ngom ueye NF Ndour CT Gueye PM Diouf M Diakhate N Long-term benefits of highly active antiretroviral therapy in Senegalese HIV-1-infected adults J Acquir Immune Defic Syndr 2005 38 14 17 10.1097/00126334-200501010-00003 15608518

[B27] Seyler C Adje-Toure C Messou E Dakoury-Dogbo N Rouet F Gabillard D Impact of genotypic drug resistance mutations on clinical and immunological outcomes in HIV-infected adults on HAART in West Africa AIDS 2007 21 1157 1164 17502726 10.1097/QAD.0b013e3281c615da PMC2486349

[B28] Tam LW Hogg RS Yip B Montaner JS Harrigan PR Brumme CJ Performance of a World Health Organization first-line regimen (stavudine/lamivudine/nevirapine) in antiretroviral-naive individuals in a Western setting HIV Med 2007 8 267 270 10.1111/j.1468-1293.2007.00463.x 17561871

[B29] Hammer SM Eron JJ Jr Reiss P Schooley RT Thompson MA Walmsley S Antiretroviral treatment of adult HIV infection: 2008 recommendations of the International AIDS Society-USA panel JAMA 2008 300 555 570 10.1001/jama.300.5.555 18677028

[B30] Garrido C Zahonero N Fernandes D Serrano D Silva AR Ferraria N Subtype variability, virological response and drug resistance assessed on dried blood spots collected from HIV patients on antiretroviral therapy in Angola J Antimicrob Chemother 2008 61 694 698 10.1093/jac/dkm515 18218644

[B31] Sungkanuparph S Manosuthi W Kiertiburanakul S Piyavong B Chumpathat N Chantratita W Options for a second-line antiretroviral regimen for HIV type 1-infected patients whose initial regimen of a fixed-dose combination of stavudine, lamivudine, and nevirapine fails Clin Infect Dis 2007 44 447 452 10.1086/510745 17205457

[B32] Cozzi-Lepri A Phillips AN Ruiz L Clotet B Loveday C Kjaer J Evolution of drug resistance in HIV-infected patients remaining on a virologically failing combination antiretroviral therapy regimen AIDS 2007 21 721 732 10.1097/QAD.0b013e3280141fdf 17413693

[B33] Mee P Fielding KL Charalambous S Churchyard GJ Grant AD Evaluation of the WHO criteria for antiretroviral treatment failure among adults in South Africa AIDS 2008 22 1971 1977 10.1097/QAD.0b013e32830e4cd8 18784460

[B34] Chaiwarith R Wachirakaphan C Kotarathititum W Praparatanaphan J Sirisanthana T Supparatpinyo K Sensitivity and specificity of using CD4+ measurement and clinical evaluation to determine antiretroviral treatment failure in Thailand Int J Infect Dis 2007 11 413 416 10.1016/j.ijid.2006.11.003 17331776

[B35] Harrigan PR Hogg RS Dong WW Yip B Wynhoven B Woodward J Predictors of HIV drug-resistance mutations in a large antiretroviral-naive cohort initiating triple antiretroviral therapy J Infect Dis 2005 191 339 347 10.1086/427192 15633092

[B36] Barth RE Wensing AM Tempelman HA Moraba R Schuurman R Hoepelman AI Rapid accumulation of nonnucleoside reverse transcriptase inhibitor-associated resistance: evidence of transmitted resistance in rural South Africa AIDS 2008 22 2210 2212 10.1097/QAD.0b013e328313bf87 18832885

[B37] Zachariah R Fitzgerald M Massaquoi M Pasulani O Arnould L Makombe S Risk factors for high early mortality in patients on antiretroviral treatment in a rural district of Malawi AIDS 2006 20 2355 2360 10.1097/QAD.0b013e32801086b0 17117022

[B38] Lawn SD Myer L Orrell C Bekker LG Wood R Early mortality among adults accessing a community-based antiretroviral service in South Africa: implications for programme design AIDS 2005 19 2141 2148 16284464 10.1097/01.aids.0000194802.89540.e1

